# Predicting Step 2 CK Performance Using Automated Feature Selection and Nested Cross-Validation

**DOI:** 10.1177/23821205261474043

**Published:** 2026-08-05

**Authors:** Padraig Mark Healy, Syed Latifi

**Affiliations:** 1 Office of Evaluation, Assessment and Student Informatics, Division of Medical Education, 36579 Weill Cornell Medicine – Qatar, Doha, Qatar

**Keywords:** nested cross-validation, machine learning, predictive model, USMLE step 2 CK, interactive dashboard

## Abstract

**Background:**

Given the recent transition of the USMLE Step 1 exam to a pass/fail system, evaluative emphasis is expected to shift toward the USMLE Step 2 Clinical Knowledge (USMLE-CK) exam, prompting the need for advanced predictive models. In this study, we proposed a multiple linear regression approach incorporating automated feature selection to predict students’ performance on the USMLE-CK exam.

**Methods:**

Our methodology integrated feature selection and model validation processes within a nested cross-validation (CV) framework to predict USMLE-CK scores. We conducted our analysis on data from four undergraduate medical student cohorts (Classes 2020 to 2023 inclusive, n = 117). A range of performance data was included for feature selection, including internal assessment data and National Board Medical Examination (NBME) *Clinical Science Subject Exams* scores. Utilizing nested CV, we constructed and assessed multiple regression models using four model evaluation metrics: mean CV error, adjusted-R^2^, Mallows’ Cp, and Bayesian Information Criteria.

**Results:**

This led to the selection of a four-predictor model (adjusted-R^2^ = 0.68). This model incorporated a combination of NBME exams (Medicine, Neurology and Surgery) and performance in a pre-clinical unit (Gastrointestinal).

**Conclusion:**

Our approach effectively streamlined the process of building a predictive model by merging feature selection with model validation. By creating an interactive, user-friendly dashboard, we empower medical educators to predict students’ USMLE-CK performance. This modeling and deployment approach holds promise for predicting student performances in other assessments.

## Introduction

The United States Medical Licensing Examination (USMLE) is a three-step licensure examination in the U.S., assessing competencies across *Step 1*, *Step 2 Clinical Knowledge (CK)*, and *Step 3*. Historically, Step 1 had a three-digit scoring system and was often used by Residency Program Directors (RPDs) to shortlist candidates.^1^ However, Step 1 transitioned to a pass/fail format in January 2022. Due to this change, the medical education community anticipated focus shifting towards USMLE-CK (which continues to provide a three-digit score) to objectively evaluate students’ caliber and suitability for residency.^2-6^ This expectation was supported by the National Resident Matching Program (NRMP)’s annual *Residency Program Director Survey*, which highlighted an increasing reliance on USMLE-CK scores as a critical component in the residency application assessment process. In 2022, 96% of RPDs considered USMLE-CK scores informative when extending interview invitations to candidates from both U.S. medical graduates (USGs) and International Medical Graduates (IMGs) applicant groups.^7,8^ In comparison to the previous years (2022 vs 2021), dependence on this rose by 2% among USGs and more pronounced 6% for IMGs. These data points not only underscore the integral role of the USMLE-CK in the residency selection landscape but also reflects the near-consensus among RPDs regarding its value as a reliable and objective measure to shortlist the number of applications submitted to their program.

The significance of this trend was further amplified in the context of the 2024 NRMP Match, with 44,853 certified applicants (highest number on record) vying for one of 41,503 PGY-1 or PGY-2 training positions amongst the 6,395 certified programs on offer.^9^ In such a competitive environment, the RPDs reliance on a quantifiable and objective metric like the USMLE-CK score has become naturally preferred, serving as a filter to manage the voluminous number of applications submitted to residency programs.

From the student’s perspective, a survey of 4,649 prospective residency applicants, including US-MD, Doctor of Osteopathic Medicine (DO), and IMG students, found that knowing the minimum USMLE-CK or Comprehensive Osteopathic Medical Licensing Examination (COMLEX) Level 2 scores required by programs is highly desired.^10^ This was especially important for IMG applicants, with 65.48% highlighting it as key, compared to 18.57% of US-MD and 15.90% of DO applicants (P<.001).^10^

Taken together, these systemic and stakeholder-level pressures create a context in which early, data-informed insights into USMLE-CK performance are increasingly valuable. This demand reflects a broader transition within higher education toward the formal adoption of learning analytics (LA) and educational data mining (EDM). LA is defined as the measurement and analysis of data regarding learners and their contexts to understand and optimize both learning and the environments in which it occurs.^11^ While these data-intensive methods are technically complex, it is essential that the primary objective of LA remains firmly grounded in the learning process itself.^12^ These practices are situated within the emerging field of educational data science, which provides a framework for integrating analytics across learning, organizations, and systems.^13^ By leveraging the methodologies of both LA and EDM, researchers can move beyond descriptive reporting to develop robust frameworks that bridge cognition and assessment.^14^ By treating institutional data not merely as a record but as a predictive asset, educators can develop models to identify students requiring academic support prior to the manifestation of performance deficits, thereby enabling more timely and targeted interventions.^15^

However, the development of such predictive models introduces additional methodological and practical considerations. A key challenge is overfitting, which can compromise the generalizability and reliability of predictions. Beyond predictive accuracy, issues of interpretability and transparency are also critical, as models must be understandable and trustworthy to support adoption by both faculty and students.^16^ Without such transparency, even high-performing models risk limited practical utility in real-world educational settings.

One commonly used tool for predicting USMLE-CK performance is the Comprehensive Clinical Science Self-Assessment (CCSSA), a practice exam that mirrors the content and structure of USMLE-CK and has been shown to correlate with exam outcomes.^17^ However, at our institution, the CCSSA is voluntary and not widely taken, resulting in limited data availability. Including it in our model would have greatly reduced the sample size. For this reason, CCSSA scores were excluded from the current analysis, though their inclusion will be revisited in future iterations of the model.

To mitigate the risk of overfitting and ensure robust model performance, we employed nested CV, in which inner folds are used for model selection and hyperparameter tuning, and outer folds are reserved for model evaluation. The aim of this study is to develop an ML model that predicts USMLE-CK scores, and to deploy it through an interactive dashboard designed for practical use by medical educators.

## Methods

### Participants

This study employed a retrospective cohort design and was conducted at Weill Cornell Medicine - Qatar (WCM-Q). WCM-Q delivers a four-year medical degree program comprised of pre-clinical (years 1 and 2), and clinical years (years 3 and 4). Pre-clinical courses consist of units that are primarily assessed via quizzes. In the clinical years, core clerkships contain clinical, custom and NBME exam components. Participants included students from Classes 2020 to 2023. The study period for these cohorts encompassed data collected from September 2016 to November 2022. A revised curriculum introduced with Class 2020 modified course structure and sequencing of certain units. This change served as a natural starting point for the study, as it marked the implementation of a consistent curriculum structure across cohorts, allowing for the collection of comparable data. However, it also restricted the sample size of this study. Inclusion criteria were: (i) enrollment in the revised curriculum, (ii) availability of complete academic performance data, and (iii) availability of USMLE-CK scores. All students meeting these criteria were included in the final analysis, with no further exclusions applied. A total of 117 students met these criteria. The final dataset comprised full data vectors, with no missing data across any of the included variables.

### Dataset

The dataset comprised first-attempt scores from pre-clinical unit quizzes, NBME subject exams, USMLE-CK, and gender. First-attempt scores were used to ensure consistency and avoid bias from repeated testing. The full list of predictor variables considered during the feature selection stage is presented in Table 1. A correlation matrix of these variables is provided in Appendix A1.

**Table 1.** Predictor Variables Considered During the Feature Selection Stage

Pre-clinical units (percent scores)	Clerkship NBME (equated percent scores)	Demographics
Biostatistics	Medicine	Gender
Brain and Behavior	Neurology	​
Cardiovascular	Ob Gyn	​
Dermatology	Pediatrics	​
Endocrinology	Primary Care	​
Gastrointestinal	Psychiatry	​
Hematology-Oncology	Surgery	​
Infectious Diseases	​	​
Pulmonary	​	​
Renal	​	​
Reproduction	​	​
Rheumatology	​	​

### Study Design

We used multiple linear regression for predicting USMLE-CK scores. We selected multiple linear regression due to its interpretability, transparency, and suitability for smaller datasets. More complex machine learning approaches were considered; however, the sample size limited the feasibility of reliably training higher-dimensional models without increasing overfitting risk. Given the small sample size, we opted for CV rather than the traditional train-test split, which typically partitions 20–30% of the data for testing.^18,19^ We adhered to a commonly endorsed guideline by maintaining a minimum of 10-15 subjects per variable (SPV) to support model stability.^20-24^ Once a model was finalized, we initiated its deployment through an interactive dashboard.

### Feature Selection (Stage-1)

This stage involved selecting variables that meaningfully contributed to the outcome while excluding those with limited explanatory value. Feature selection was performed using the regsubsets() function from the [leaps package] in R^25^ to select the best models based on the Residual Sum of Squares (RSS). In this function, we specified a maximum of five features to include in the model, which resulted in the algorithm returning five models: the best one-variable model, the best two-variable model, and so on, up to the best five-variable model. The limit was chosen to ensure that the largest model produced (5 variable), maintained the 10-15 SPV rule of thumb.^20-24^

To identify the optimal combination of variables needed to predict a student’s USMLE-CK performance, an exhaustive best subset selection technique was used. This method evaluated all possible combinations of predictors and found the best subset according to the chosen criteria. The regsubsets() function generated a summary table that displays the best n feature variable models and is presented in Appendix A2.

### Model Validation (Stage-2)

To evaluate model performance, we used K-fold CV (k=3). The value of k was selected to ensure a reasonable number of data points per fold, given the limited sample size. The validation process was repeated three times to enhance the stability of the results. In each iteration, two folds were used as the training-set, and one as the test-set. Next, models were fitted on the training data and evaluated on the test data. The accuracy of each iteration was computed, and the estimated error rate was derived as the average error rate across the three iterations. This approach made efficient use of the limited sample size, while minimizing overfitting.

### Nested CV

**Figure 1.**
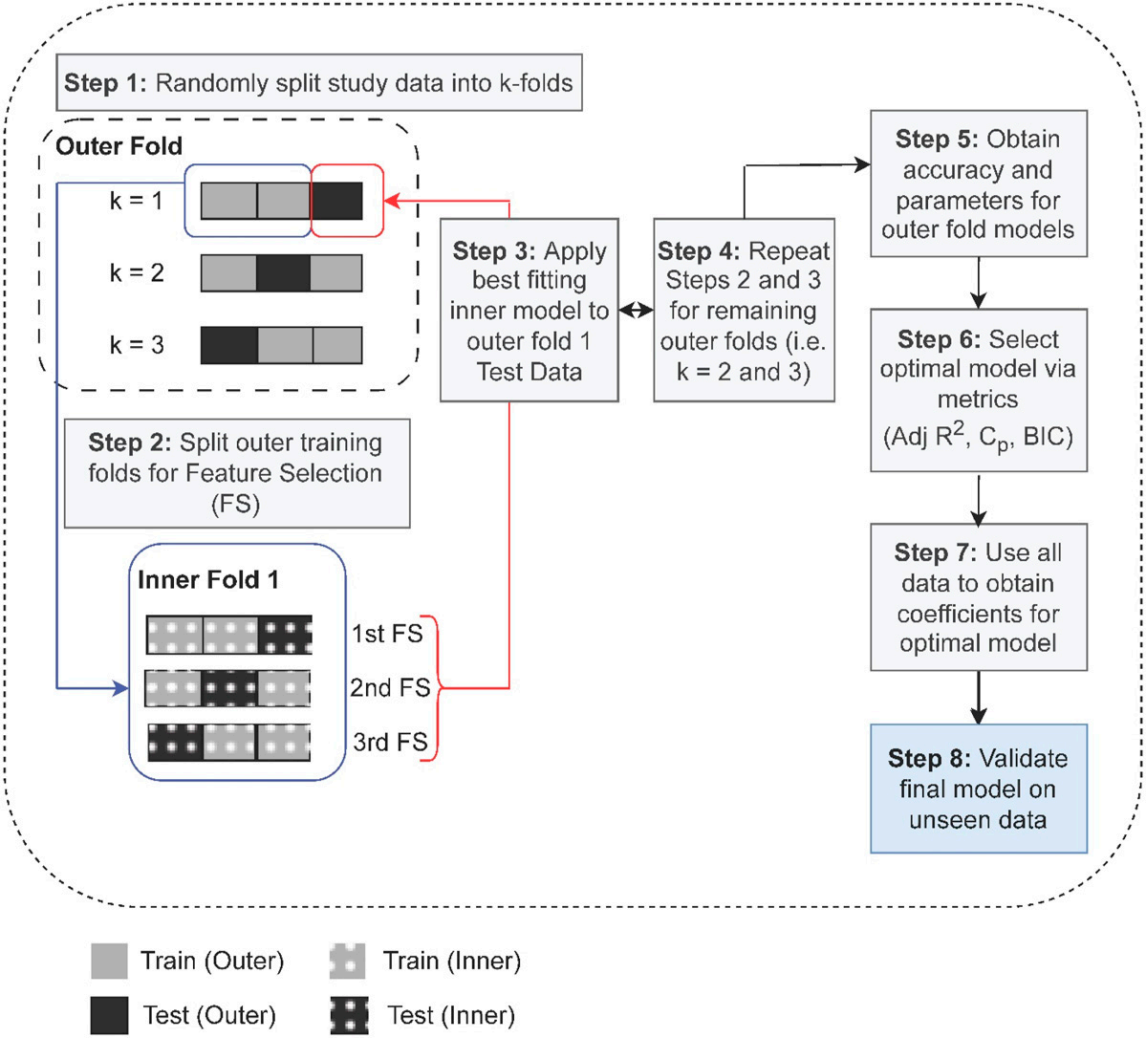
Nested CV process*. *Step 1: Split the data into training and test data groupings (k = 3 in this application). Step 2: For each of the outer training folds, split them into inner folds for feature selection. Step 3: In this inner fold, select the best model and use it to test the outer fold. Step 4: Repeat steps 2 and 3 for the remaining outer folds. Step 5: Select the best outer models. Step 6: Select the optimal model based on metrics. Step 7: Use/apply the selected model to the entire dataset to obtain coefficients. Step 8: Validate the model on unseen data

This approach combined Stage-1 and -2 and is recommended over the train-test approach in many instances.^26,27^ The order in which these two stages are executed is crucial. Were we to apply the feature selection algorithm on the entire dataset before carrying out CV, it could have led to positive bias, meaning the feature selection should occur nested within the CV.^28,29^ Figure 1 illustrates the nested CV framework.

### Sensitivity Analysis: Bootstrapping for Variable Selection Stability

To further evaluate the robustness of the four-predictor model and mitigate possible concerns regarding overfitting, we conducted a variable stability analysis using a bootstrapping approach.^16,30^ This moved beyond point estimates towards selection frequency to determine how consistently the algorithm identified each predictor across resampled versions of the data.

We executed 5,000 bootstrap iterations (with replacement). For each iteration, the exhaustive all-subsets selection algorithm was tasked with identifying the optimal four-variable model. This enabled us to calculate a selection frequency for each variable, representing the percentage of bootstrap runs in which a specific predictor was included in the final set. We subsequently cross-referenced these frequencies with the correlation matrix (Appendix A1) to identify potential sources of instability such as multicollinearity.

### Dashboard Design and Development

Having developed a predictive model, our focus now shifted toward its deployment. We contended that for a model to possess true utility, it had to excel in two key areas: (i) predictive utility (i.e., how accurate it was at predicting scores) and (ii) end-user utility (i.e., ease of use and accessibility). To support practical implementation, we engaged stakeholders early in the design process to prioritize usability alongside predictive accuracy.

After eliciting stakeholder feedback, the next stage was ‘storyboarding’, in which the designers built a prototype to share with stakeholders for more feedback and refinement. To initiate this process, we convened with stakeholders to establish and prioritize requirements. The objective of this dashboard was to facilitate the prediction of students’ performances on the USMLE-CK exam. However, the dashboard designers sought additional information from stakeholders on what a fit-for-purpose dashboard should include from their perspectives as subject-matter experts. We suggested that they apply the MoSCoW prioritization framework^31^ and designate all possible features into one of four categories: *‘must have’*, *‘should have’*, *‘could have’* or *‘will not have’*. This collaborative process ensured that the dashboard was both functional and aligned with user needs.

In implementing the model within Power BI, we utilized the SELECTEDVALUE function to retrieve user-entered values for the predictive variables. These values were then incorporated into the regression model within Power BI, enabling dynamic calculation of a student’s predicted score in a user-friendly manner. The predicted scores were categorized into quartiles using the PERCENTILEX.EXC function. This helped provide a comparative analysis of a student’s performance relative to their peers at WCM-Q. The outcome of this design process is described in the results section and an extract of the dashboard is provided in Figure 3.

### Software Tools

All statistical analyses and model development were conducted using R (version 4.2.2),^25^ including feature selection, CV procedures, model evaluation plots and bootstrapping. The dashboard was developed using Microsoft Power BI.^32^

### Ethical Considerations

In line with institutional regulatory guidance, this work was determined by our Institutional Review Board (IRB) to align with program-level quality improvement and therefore did not require formal IRB oversight. Data was collected and managed in accordance with guidelines to ensure confidentiality. All data were utilized solely for the purpose of improving program quality.

## Results

### Feature Selection (Stage-1)

A four-variable model emerged as the preferred choice for accurate USMLE-CK performance prediction. This model was selected based on four evaluation metrics: mean CV error, adjusted-R^2^, Mallow’s Cp, and Bayesian Information Criteria (BIC).

**Figure 2.**
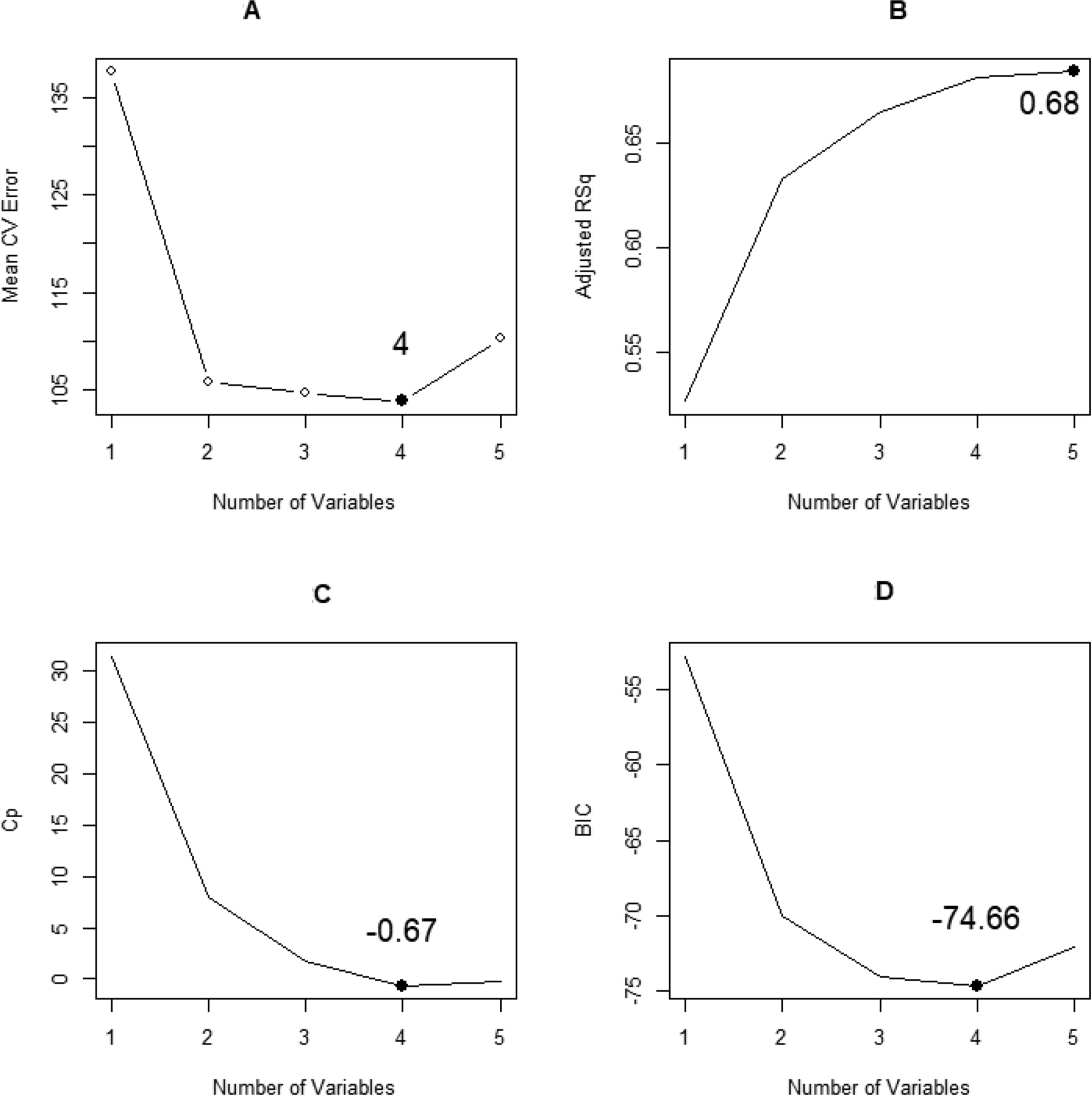
Metrics for selecting number of features in model: (A) Mean Cross-Validation (CV) Error, (B) Adjusted-R^2^, (C) Mallows Cp, (D) Bayesian Information Criteria (BIC)

The mean CV error plot (Figure 2A) illustrates how predictive performance changes as the numbers of variables increases. The optimal model, balancing predictive accuracy and model complexity, was indicated by the lowest point on this plot. Upon examining the mean CV error, we observed an improvement in the model’s fit with the addition of the initial variables. However, after a certain number of variables were incorporated, the model’s efficiency began to plateau. This reduction in efficiency became evident upon the inclusion of the fifth variable, i.e., the model reached an optimal level of efficiency at the fourth variable.

Similarly, the adjusted-R^2^ plot (Figure 2B) reflects the balance between model complexity and explanatory power (i.e. variance explained by the predictors). A higher adjusted-R^2^ value signified a more accurate model fit. We observed that the initial inclusion of more variables led to an increase in the adjusted-R^2^ value, indicating a rise in the model’s explanatory power. However, similar to the mean CV error plot, once the model exceeded a certain number of variables, the adjusted-R^2^ value began to decline, signifying that additional complexity no longer contributed to an increase in explanatory power.

Mallow’s Cp and the BIC (Figure 2C and D) served as model selection metrics that assessed the trade-off between a model’s fit to the data and its complexity. BIC, in particular, imposed a greater penalty for adding variables than Mallow’s Cp, promoting a more judicious balance between fitting precision and simplicity. The ideal model, according to both metrics, was denoted by the lowest possible values. The initial addition of variables reduced the values of both Mallow’s Cp and BIC, which indicated a model with a strong fit and low complexity. However, as more variables were included, these values began to rise, suggesting that any further complexity could have led to diminishing benefits and a risk of overfitting the model to the data.

While both the four- and five-predictor models achieved the same adjusted-R^2^ of 0.68 (rounded to two decimal places), the four-predictor model was selected as the more parsimonious option, and it also aligned with the other three model evaluation criteria. Bootstrap resampling (5,000 iterations) was used to assess the sampling variability of the adjusted-R^2^ for the final four-predictor model. The mean adjusted-R^2^ was 0.64, with a 95% confidence interval (CI) ranging from 0.52 to 0.76.

### Model Validation (Stage-2)

With the model size decided upon, the next step was to obtain the coefficients. To do this, the best subset selection was conducted on the full data and the best four-variable model was chosen. This resulted in the selection of three clerkship NBME exam first time performance (Medicine, Neurology, and Surgery), and one pre-clinical unit quiz performance: (Gastrointestinal). The resulting prediction equation was: Predicted USMLE-CK score = 58.87 + 0.44 (Medicine) + 0.80 (Neurology) + 0.47 (Surgery) + 0.59 (Gastrointestinal). Model performance yielded a mean square error (MSE) of 177.96. This value indicates the model’s generalization ability on unseen data.

The selected predictor set reflects the content distribution and construct alignment of the USMLE Step 2 CK examination. Consistent with this, prior work has demonstrated that NBME subject examination performance is among the strongest predictors of Step 2 CK outcomes, with particularly robust effects observed for core clerkship examinations.^2^ In particular, the inclusion of internal medicine is consistent with the Step 2 CK blueprint, in which internal medicine represents the largest proportion of assessed content, accounting for approximately 55–65% of test material.^33,34^ The inclusion of the Gastrointestinal unit (the sole pre-clinical predictor) represents the foundational knowledge base required for subsequent clinical integration. While this variable exhibits high multicollinearity with other organ-system units (e.g., Renal, *r* = 0.75; Cardiovascular, *r* = 0.69), its selection in the optimal model suggests it serves as a parsimonious proxy for general pre-clinical academic standing. This is consistent with validity evidence indicating that Step 2 CK success is predicated on the ability to apply foundational science concepts to clinical care.^35^

### Model Comparison: Nested CV vs Train-Test

Next, we compared the nested CV model’s predictive performance to a more traditional modelling approach (train-test) using MSE values. For the train-test method, the data was randomly partitioned: 70% for training and 30% for testing. The same four-variable model, identified through the nested CV process, was applied in the train-test approach to ensure a like-for-like comparison. Predicted scores were then computed for each method, and MSE values were calculated.

The MSE values in Table 2 reflect differences in prediction accuracy across different data samples and modelling methods. A lower MSE suggested that predicted values were close to observed values on average, indicating better model performance. The MSE obtained from nested CV was 177.96, with a 95% CI of 67.30 to 332.60, should be interpreted as a realistic estimate of generalization error and is most appropriately compared to the test-set MSE, not the train-set MSE. The training MSE was 72.03 (95% CI: 46.18 to 92.62) reflects the model’s performance on data it was trained on and is therefore optimistically biased. The test set MSE was higher at 188.37 (95% CI: 164.30 to 241.44), reflecting the model’s reduced predictive accuracy on unseen data. The overlap between the nested CV and test-set CI’s suggest that the model’s performance under both evaluation methods may be comparable. However, the wide interval for the nested CV estimate reflects greater variability, and uncertainty due to resampling.

**Table 2.** Comparison of Model Performance Using MSE

Evaluation method	MSE	95% CI
Nested CV	177.96	67.30 – 332.60
Train	72.03	46.18 – 92.62
Test	188.37	164.30 – 241.44

### Sensitivity Analysis: Bootstrapping for Variable Selection Stability

To assess the robustness of our four-predictor model against overfitting, we performed a 5,000-iteration bootstrap stability analysis.^30^ By identifying the best four predictors for each resample, we calculated selection frequencies to quantify the signal strength of each possible predictor variable.

The results revealed a hierarchy of stability amongst the predictors. Medicine (65.78%) and Neurology (65.60%) emerged as highly robust, appearing in almost two-thirds of all iterations. These frequencies exceed the ≥60% threshold proposed by Austin and Tu^36^ for identifying stable predictors in parsimonious models, suggesting these variables are reliable indicators of performance. In contrast, Surgery appeared less often (37.56%); however, its selection frequency was likely suppressed by its high correlation with Medicine (r = 0.68), leading to competitive selection between the two. Similarly, the Gastrointestinal unit demonstrated low stability (6.98%), a result of multicollinearity with other pre-clinical units, including Renal (*r* = 0.75), Pulmonary (*r* = 0.73) and Cardiovascular (*r* = 0.69). This can cause the algorithm to alternate between redundant features during resampling. Despite its instability, Gastrointestinal was retained because it provided the optimal balance of fit and parsimony on the full dataset.

### Dashboard Implementation

**Figure 3.**
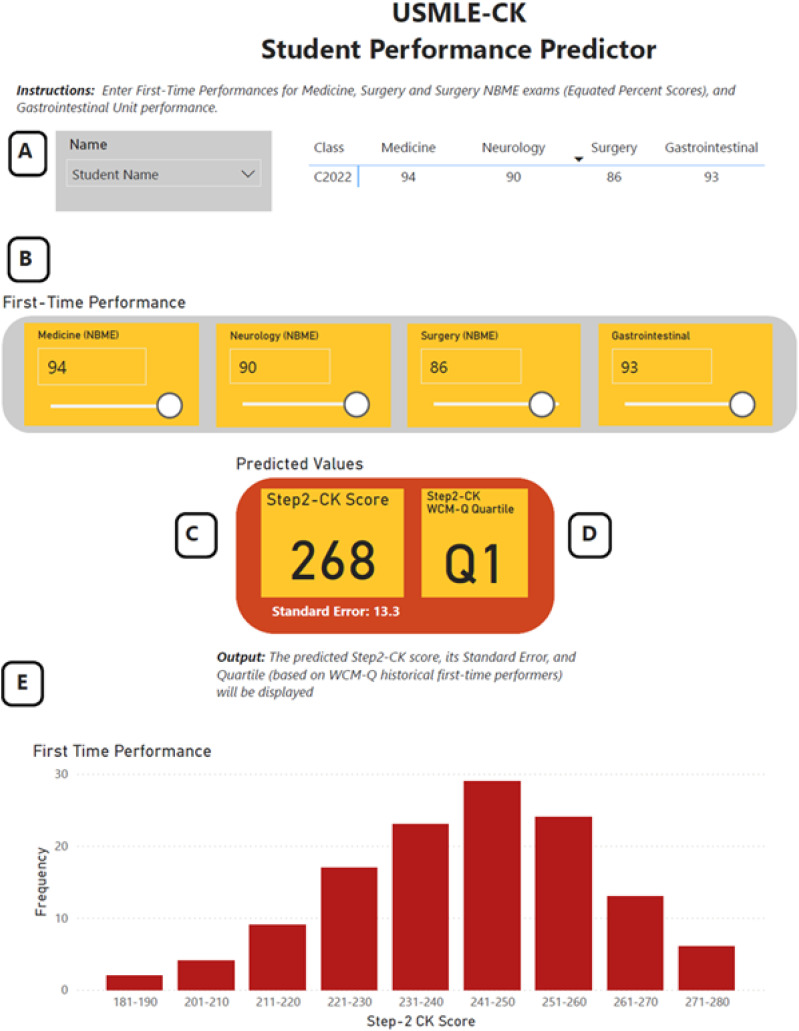
Step 2 CK student performance predictor dashboard

The design discussions with stakeholders for the dashboard’s components led to the decision to incorporate five key features (see Figure 3A–E), each chosen for their specific utility in enhancing the predictive and user experience aspects of the tool (i.e., empowering users to interact with the model). These features included, (a) a student name drop-down list for enabling retrieval of their scores, (b) input fields for the variables required to predict USMLE-CK performance - as identified by our analysis, (c) predicted USMLE-CK score with standard error, with the latter reflecting the uncertainty in the prediction, indicating expected variability across different samples^37^ (d) predicted quartile (program-level), and (e) a histogram displaying the distribution of historical students’ first-time performances on the exam. The latter two features provided generalizable interpretability, i.e., they enabled the user to gauge the level of risk with a student by benchmarking them against the program’s historical data (quartile and histogram). In addition to complementing the aforementioned measures (c – d), the histogram also provided a visual insight into the skewness and spread of student performances, as well as highlighting outliers. Overall, the dashboard provided a user-friendly, data-driven tool for interacting with the model.

## Discussion

This study highlights two key contributions: (i) the use of a nested CV framework to develop a robust predictive model despite a limited sample size, and (ii) the deployment of this model through a dashboard that enables educators to make informed, data-driven decisions. These strengths address challenges faced in medical education research including data sparsity and the need for practical, accessible tools for faculty.

The traditional train-test split approach, though common, is not without its limitations, such as risks of overfitting and producing unreliable estimates.^38^ In contrast, our method is less susceptible to overfitting and remains robust in small sample size contexts.^26,27^

Another contribution of this study was the operationalizing of the model through an interactive dashboard. This dynamic tool provides medical educators with real-time access to predictive insights and supports its integration into academic advising workflows. The model offers two primary use cases. First, it can assist educators in advising students on residency planning, especially as USMLE-CK scores are gaining prominence in residency interview selection decisions.^2-6^ A predictive tool for USMLE-CK scores could help medical educators guide students in aligning their applications with their academic profiles. Second, the model can aid identifying students who may be academically at risk, enabling early and targeted intervention. However, as observed by Shanks,^15^ the disclosure of such predictions can influence student motivation and anxiety. Therefore, caution should be taken to use the dashboard as a starting point for holistic academic support, rather than an isolated metric.

Despite these strengths, several limitations should be acknowledged. First, the relatively small sample size may have constrained the models’ robustness. Additionally, the specific characteristics of the included cohorts may limit how broadly the findings can be applied. Furthermore, as the data was derived from a singular institution, the model reflects a specific curricular structure, and as such, the generalizability of the model to other institutional contexts, curriculum, or assessment practices remains uncertain^39,40^. This study also did not control for socio-demographic factors such as age, race, ethnicity, and social class, which could have affected the generalizability of the results.^41^ The exclusion of CCSSA scores (due to their limited availability at our institution) represented an additional limitation, as these scores are known to correlate with USMLE-CK outcomes.^17^

In addition, although nested cross-validation was employed to mitigate overfitting, the combination of a small sample and single-institution dataset introduces uncertainty in model performance estimates. Cross-validation results in small samples are known to exhibit high variance and narrow error estimates, limiting the reliability of reported performance.^42^ Furthermore, it is important to acknowledge that adjusted-R^2^ estimates can remain optimistically biased in small sample sizes.^21^ In this study, the adjusted-R^2^ of the final four-predictor model was 0.68, whereas bootstrap resampling produced a mean adjusted-R^2^ of 0.64 (95% CI: 0.52 to 0.76). The reduction and variability observed across resamples suggest that the model’s explanatory performance is sensitive to sampling variation.

Future work will involve implementing a cyclical recalibration process for the algorithm as new cohort data becomes available. This measure is designed to mitigate a phenomenon known as model drift, where the model’s predictive power diminishes over time due to shifts in the underlying data. Another important future step is external validation using data from other institutions to assess generalizability across differing educational contexts and learner populations.^43^ Such extensions should ideally include prospective or multi-institutional evaluation to further examine whether model performance is sustained under varying institutional settings. Furthermore, this methodology could be extended to construct and deploy similar predictive models for high-stakes assessments within the educational program.

## Conclusion

This study detailed the development of a model for predicting USMLE-CK performance, leveraging nested CV to maintain methodological rigor despite a limited sample size. The deployment of the model through an interactive dashboard marks another contribution, turning institutional data into a predictive asset, readily accessible to medical educators and advisors. This approach also provides an adaptable framework for other constructing and implementing predictive models for other high-stakes assessments in medical education.

## Data Availability

The data used in this study are available from the corresponding author upon a reasonable request.*

## Appendix

**Appendix A1:** Correlation Matrix

​	Biostats	CVS	Pulmonary	GI	Renal	Hemeonc	BAB	Endocrine	Derm	ID	Rheum	Repro	Medicine	Neurology	Surgery	Ob.Gyn	Pediatrics	Primary.Care	Psychiatry	Step2_CK
Biostats	1	​	​	​	​	​	​	​	​	​	​	​	​	​	​	​	​	​	​	​
CVS	0.7	1	​	​	​	​	​	​	​	​	​	​	​	​	​	​	​	​	​	​
Pulmonary	0.54	0.73	1	​	​	​	​	​	​	​	​	​	​	​	​	​	​	​	​	​
GI	0.64	0.69	0.73	1	​	​	​	​	​	​	​	​	​	​	​	​	​	​	​	​
Renal	0.59	0.68	0.7	0.75	1	​	​	​	​	​	​	​	​	​	​	​	​	​	​	​
HemeOnc	0.61	0.65	0.66	0.62	0.68	1	​	​	​	​	​	​	​	​	​	​	​	​	​	​
BAB	0.7	0.71	0.72	0.73	0.75	0.68	1	​	​	​	​	​	​	​	​	​	​	​	​	​
Endocrine	0.55	0.61	0.63	0.66	0.68	0.5	0.7	1	​	​	​	​	​	​	​	​	​	​	​	​
Derm	0.5	0.46	0.51	0.53	0.55	0.47	0.69	0.54	1	​	​	​	​	​	​	​	​	​	​	​
ID	0.46	0.49	0.55	0.45	0.5	0.58	0.62	0.44	0.66	1	​	​	​	​	​	​	​	​	​	​
Rheum	0.55	0.55	0.55	0.56	0.69	0.57	0.78	0.52	0.64	0.62	1	​	​	​	​	​	​	​	​	​
Repro	0.5	0.5	0.43	0.42	0.49	0.56	0.53	0.37	0.47	0.49	0.53	1	​	​	​	​	​	​	​	​
Medicine	0.52	0.62	0.56	0.56	0.61	0.58	0.57	0.51	0.41	0.51	0.47	0.44	1	​	​	​	​	​	​	​
Neurology	0.45	0.55	0.54	0.53	0.59	0.46	0.54	0.57	0.41	0.49	0.42	0.4	0.64	1	​	​	​	​	​	​
Surgery	0.48	0.55	0.55	0.54	0.63	0.46	0.55	0.53	0.33	0.46	0.49	0.34	0.68	0.62	1	​	​	​	​	​
Ob.Gyn	0.41	0.44	0.58	0.57	0.65	0.49	0.6	0.48	0.54	0.41	0.53	0.42	0.53	0.59	0.62	1	​	​	​	​
Pediatrics	0.52	0.58	0.56	0.54	0.61	0.48	0.62	0.6	0.48	0.49	0.5	0.53	0.67	0.6	0.58	0.6	1	​	​	​
Primary.Care	0.42	0.43	0.46	0.39	0.47	0.48	0.47	0.42	0.34	0.4	0.39	0.42	0.62	0.47	0.57	0.44	0.59	1	​	​
Psychiatry	0.4	0.47	0.56	0.56	0.52	0.37	0.53	0.52	0.45	0.39	0.47	0.4	0.59	0.57	0.55	0.59	0.66	0.48	1	​
Step2_CK_Score	0.5	0.5	0.5	0.55	0.59	0.47	0.59	0.51	0.46	0.57	0.46	0.46	0.72	0.69	0.68	0.58	0.63	0.61	0.64	1

**Appendix A2:** Subset Selection of Best n Feature(s)

N predictor(s)	...	...	...	Gastrointestinal	...	...	...	...	...	Medicine	Neurology	Surgery
1	...	...	...	—	...	...	...	...	...	*	—	—
2	...	...	...	—	...	...	...	...	...	*	*	​
3	...	...	...	—	...	...	...	...	...	*	*	*
4	...	...	...	*	...	...	...	...	...	*	*	*
...	...	...	...	...	...	...	...	...	...	...	...	...
19	*	*	*	*	*	*	*	*	*	*	*	*

*Indicates feature selected at the n^th^ predictor.

### List of Abbreviations

BIC: Bayesian Information Criteria
CCSSA: Comprehensive Clinical Science Self-Assessment
CI: Confidence Interval
COMLEX: Comprehensive Osteopathic Medical Licensing Examination
CV: Cross-validation
DO: Doctor of Osteopathic Medicine
EDM: Educational Data Mining
IMGs: International Medical Graduates
IRB: Institutional Review Board
LA: Learning Analytics
MD: Doctor of Medicine
ML: Machine learning
MSE: Mean Squared Error
NRMP: National Resident Matching Program
PG: Postgraduate
RPD: Residency Program Director
RSS: Residual Sum of Squares
SPV: Subjects per variable
USMLE-CK: United States Medical Licensing Examination Step 2 Clinical Knowledge
USGs: United States Medical Graduates
USMLE: United States Medical Licensing Examination.
WCM-Q: Weill Cornell Medicine - Qatar
